# Complete Response to Cetuximab Plus Paclitaxel Therapy in Nivolumab-Refractory Patients in Distant Metastasis of Squamous Cell Carcinoma of the Tongue: A Report of Two Cases

**DOI:** 10.7759/cureus.49198

**Published:** 2023-11-21

**Authors:** Hidetake Tachinami, Kei Tomihara, Danki Takatsuka, Atsushi Ikeda, Shin-ichi Yamada, Makoto Noguchi

**Affiliations:** 1 Oral and Maxillofacial Surgery, Toyama University, Toyama, JPN; 2 Oral and Maxillofacial Surgery, Graduate School of Medical and Dental Sciences, Niigata University, Niigata, JPN

**Keywords:** case report, cetuximab, paclitaxel, distant metastatic oral squamous cell carcinoma, nivolumab-refractory

## Abstract

Herein, we report two cases of patients diagnosed with nivolumab-refractory distant metastatic squamous cell carcinoma of the tongue who were successfully treated with a combination of paclitaxel and cetuximab. Case 1 had controllable local recurrence and distant metastasis. Case 2 had controllable distant metastatic disease. Thus, demonstrating that some nivolumab-refractory patients with recurrent or distant metastatic oral squamous cell carcinoma may benefit from subsequent salvage chemotherapy.

## Introduction

Nivolumab, an immune checkpoint inhibitor (ICI), has revolutionized the treatment of recurrent and metastatic oral cancer [1]. Nivolumab is a human monoclonal IgG4 antibody that targets the negative immunomodulator PD-1 on T-cells. It was approved in Japan in 2017 and has since been used to treat advanced oral cancer. However, the overall response rate (ORR) in patients with advanced oral cancer is less than 20% [1]. Though it has been well-documented in the literature that conventional chemotherapy is highly effective for patients with advanced lung cancer in conjunction with nivolumab administration, only a few reports of its use in head and neck cancer are available [2]. In this study, we report two cases of patients with tongue cancer who presented with progressive disease after nivolumab administration and subsequently responded to salvage chemotherapy. Although there is prolonged overall survival with salvage chemotherapy, a complete response is rare. We report a complete response to cetuximab plus paclitaxel after chemotherapy in two patients with distant metastases to the liver and bone. This article was previously posted to the AUTHOREA preprint server on March 9, 2022.

## Case presentation

Case 1

A 43‐year‐old male patient (height: 171cm; weight: 79kg; with good nutritional status) presented to our hospital with an ulcer on the left tongue margin. He had no prior relevant medical history, did not consume alcohol or smoke, and had no family history of cancer. He appeared to be well nourished. While there were no significant extraoral findings, intraoral examination revealed a raised, ulcerated lesion measuring 40 × 30 mm that extended from the left lateral margin to the ventral surface of the tongue.

**Figure 1:**
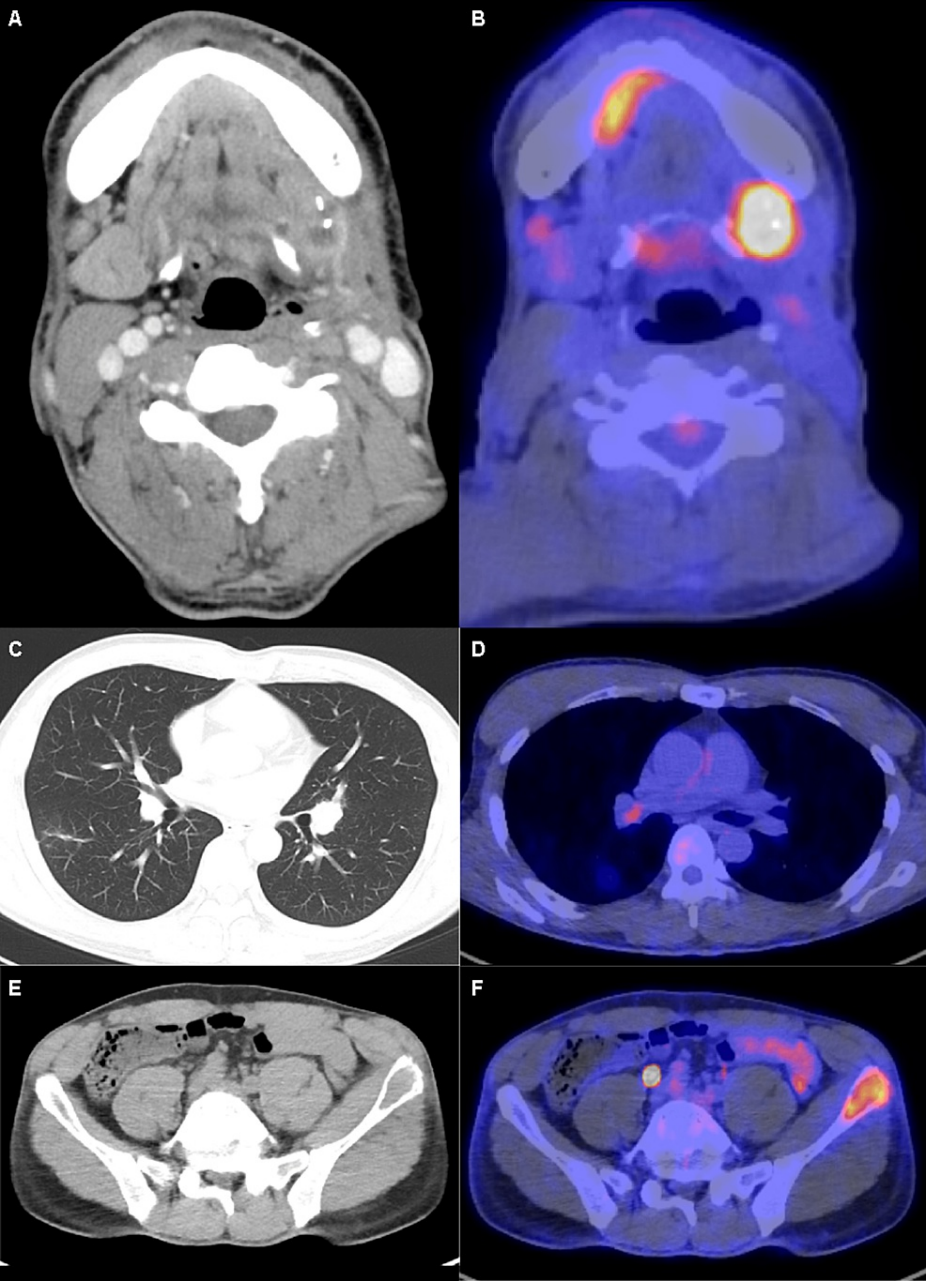
Radiological findings of the tumor in Case 1 before nivolumab administration. Findings before nivolumab administration. Computed tomography (CT; left) revealed a swelling in the left submandibular region (A, B), pale ring-shaped nodular shadows in both the lung fields (C, D), and osteolytic changes in the left iliac bone (E). Fluorodeoxyglucose-positron emission tomography (FDG-PET; right) revealed an abnormal accumulation in the left iliac bone (F).

**Figure 2:**
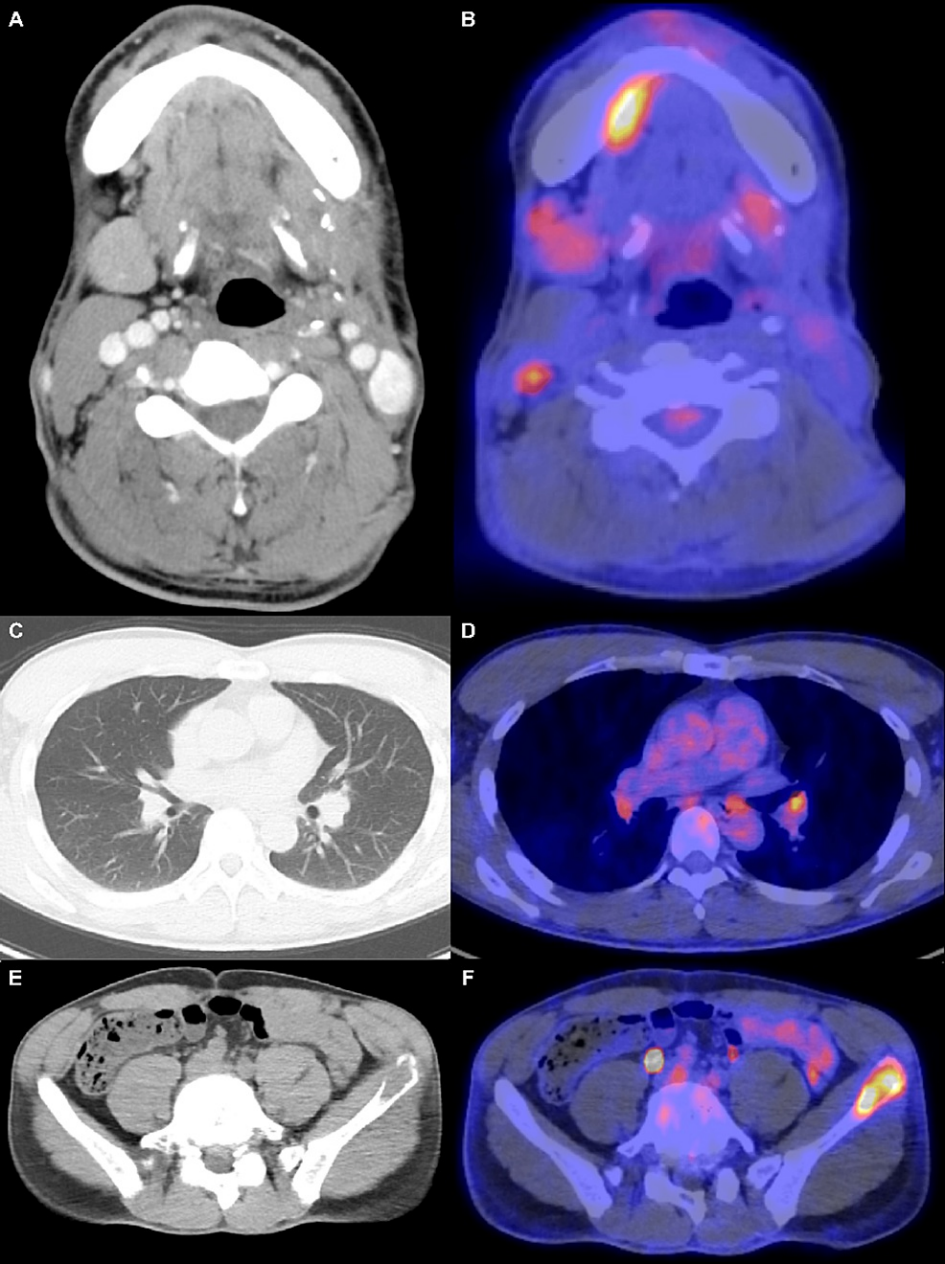
Radiological findings of the tumor in Case 1 after nivolumab administration. Findings after 10 courses of nivolumab administration. Contrast-enhanced CT and FDG-PET revealed enlargement of each target lesion, indicating progressive disease (A-F).

**Figure 3:**
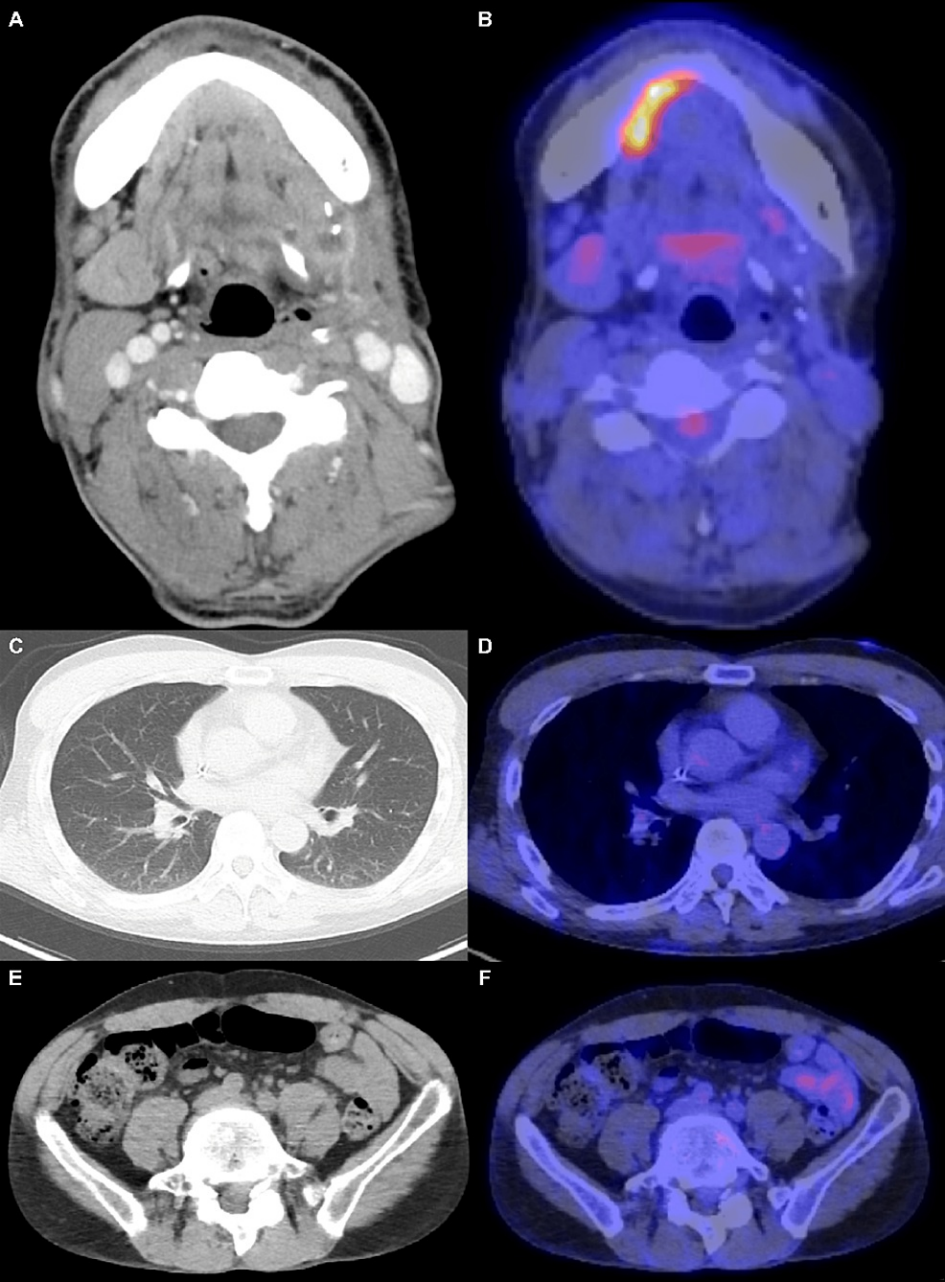
Radiological findings of the tumor in Case 1 after salvage chemotherapy. After 16 courses of salvage chemotherapy, all target lesions almost disappeared, indicating a complete response (A-F). As suggested, we have improved.

The histological diagnosis of the biopsy specimen was squamous cell carcinoma; the patient was diagnosed with squamous cell carcinoma of the tongue (T3N0M0, Stage III). After platinum-based chemotherapy, the patient underwent neck dissection, partial left-sided tongue resection, and anterolateral thigh flap reconstruction. Eight months later, computed tomography (CT) images revealed a swelling in the left submandibular region, pale ring-shaped nodular shadows in both lung fields, and osteolytic changes in the left iliac bone (Figures 1A, 1C, 1E). Fluorodeoxyglucose-positron emission tomography (FDG-PET) revealed an abnormal accumulation in the left submandibular region, both lung fields, and left iliac bone (Figures 1B, 1D, 1F). In view of these findings, nivolumab (3 mg/kg) was administered every two weeks. After completion of 10 cycles, contrast-enhanced CT and FDG-PET revealed enlargement of each target lesion, indicating progressive disease (iliac bone metastasis and left lung lesion) (Figures 2A-2F). The patient was started on paclitaxel and cetuximab (PC) therapy (paclitaxel: 80 mg/m2, cetuximab: 400 mg/m^2^). After 16 courses of PC therapy, all target lesions had decreased in size, indicating a partial response. After 46 courses of PC therapy, all target lesions had almost disappeared, indicating a complete response (Figures 3A-3F). There was no recurrence after 36 months. 

Grade 1 hypothyroidism, which occurred as a secondary immune-related adverse event, was treated with levothyroxine sodium hydrate (Tirazin®) at the Endocrinology Department of our hospital. After PC therapy, the patient developed grade 2 interstitial pneumonia; thus, steroid therapy was initiated, which required hospitalization for approximately 1 week. In addition, he developed grade 2 dermatitis. However, no secondary symptoms of grade 3 or higher levels of dermatitis were observed.

Case 2

A 67‐year‐old female patient (height: 148cm; weight: 59kg; with good nutritional status) presented to our hospital with an ulcer on the right tongue margin. The patient’s hematological findings at the initial examination revealed a hemoglobin Alc level of 6.0%. She did not consume alcohol or smoke and had no family history of cancer. Extraoral examination revealed no significant findings; however, intraoral examination revealed a raised, ulcerated lesion 30 × 30 mm in size extending from the right lateral margin to the ventral surface of the tongue.

**Figure 4:**
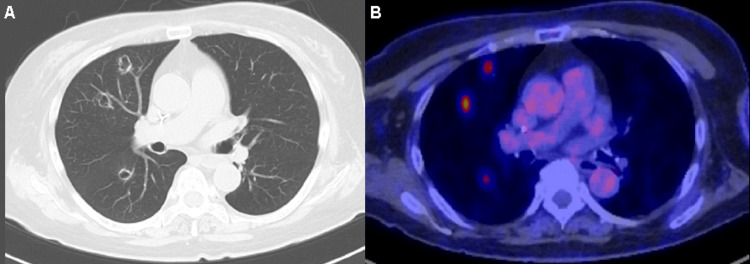
Radiological findings of the tumor in Case 2 before nivolumab administration. Findings before nivolumab administration. Computed tomography (CT; A) and fluorodeoxyglucose positron-emission tomography (FDG-PET; B) revealed multiple cavernous nodules in the right lung field.

**Figure 5:**
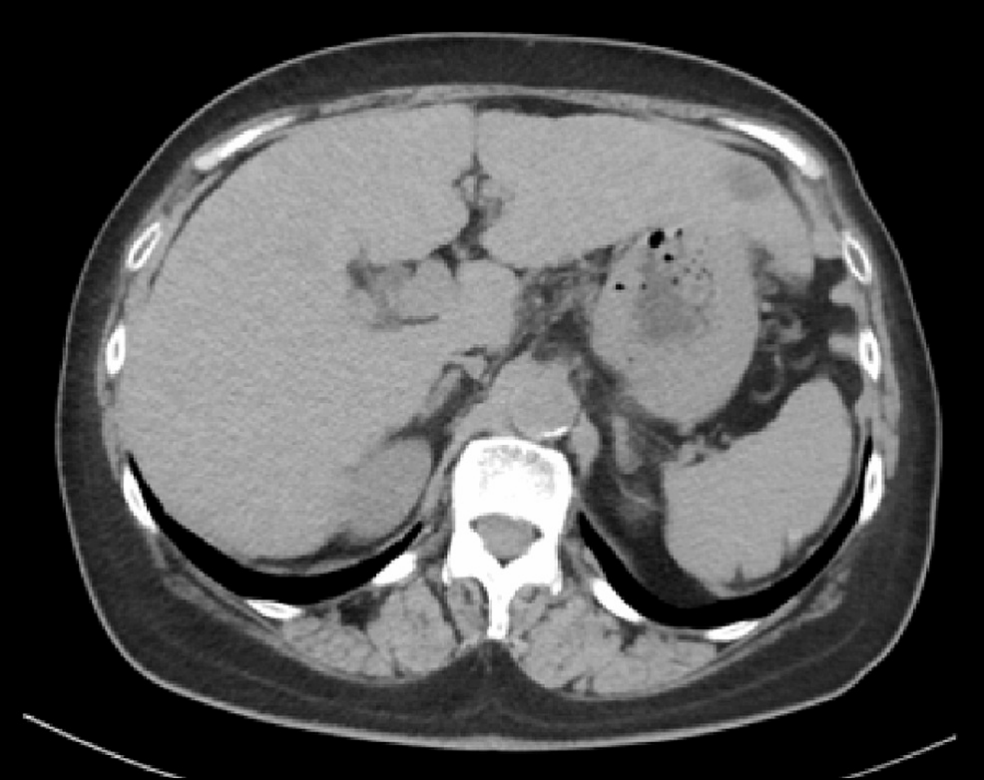
Radiological findings of the tumor in Case 2 after nivolumab administration. After nine courses of nivolumab, contrast-enhanced CT revealed an increase in the size of the target lesion and a new low-density nodule in the left lateral lobe of the liver.

**Figure 6:**
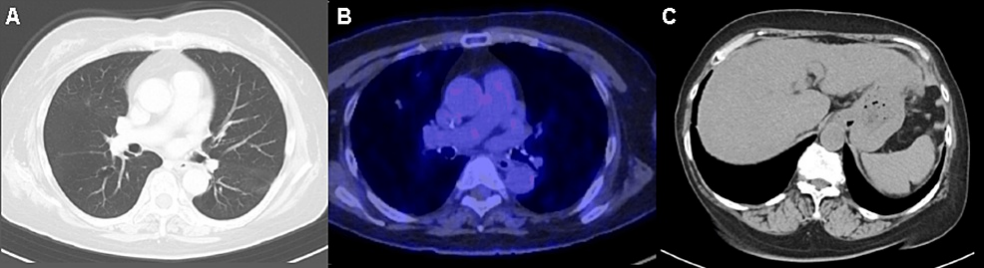
Radiological findings of the tumor in Case 2 after salvage chemotherapy. After 19 courses ofsalvage chemotherapy, all target lesions almost disappeared, which indicated a complete response (A-C).

The histological diagnosis of the biopsy specimen was squamous cell carcinoma; the patient was diagnosed with squamous cell carcinoma of the tongue (T3N0M0, Stage III). The patient refused surgery and received chemoradiation therapy. Three months later, the primary tumor was under control; however, CT and FDG-PET revealed multiple cavernous nodules in the right lung field (Figures 4A, 4B). The patient was started on nivolumab (3 mg/kg, administered every two weeks) for multiple lung metastases. After nine courses of nivolumab, contrast-enhanced CT revealed an increase in the size of the target lesion and a new low-density nodule in the left lateral lobe of the liver, which was diagnosed as liver metastasis (Figure 5). The patient was started on PC therapy (paclitaxel: 80 mg/m^2^, cetuximab: 400 mg/m^2^). After 19 courses of PC therapy, all target lesions had almost disappeared, which indicated a complete response (Figures 6A-6C). No recurrence was observed at the 36-month follow-up.

Grade 1 hypothyroidism, which occurred as a secondary immune-related adverse event, was treated with levothyroxine sodium hydrate (Tirazin®) at the Endocrinology Department of our hospital. Following PC therapy, the patient developed grade 2 liver function abnormality and grade 2 dermatitis, which were treated with ursodeoxycholic acid (Urso®️) at the Department of Gastroenterology. However, no severe secondary symptoms of grade 3 or higher levels of dermatitis were observed.

## Discussion

Nivolumab, an ICI that blocks programmed death-1 (PD-1; an immune checkpoint molecule), was approved in 2017 in Japan as a treatment option for recurrent or distant metastatic advanced oral cancer. Nivolumab has brought about a revolution in the treatment of oral cancer by significantly improving patients’ prognoses. However, its efficacy is limited. The ORR is low, and additional salvage therapy and subsequent chemotherapy are required for refractory patients. Although there is prolonged overall survival with salvage chemotherapy, a complete response is rare. We report a complete response to cetuximab plus paclitaxel after chemotherapy in two patients with distant metastases in the liver and bone.

Following the advent of ICIs in recent years, salvage chemotherapy has been found to be effective for several solid tumors, including those in patients with head and neck cancer [2-5]. Because ICIs targeting PD-1 occupy T cells for over two months, the effects of salvage chemotherapy may overlap those of prior nivolumab therapy. Nivolumab reportedly inhibits the binding of programmed death ligand-1 (PD-L1; expressed on cancer cells) to the PD-1+ CD8+ T cells in the tumor microenvironment, thereby inhibiting PD-1/PD-L1 signaling [6]. However, in the actual tumor microenvironment, immunosuppressive cells, such as regulatory T cells (Tregs) and myeloid-derived suppressor cells (MDSCs), prevent the nivolumab-mediated antitumor immune response [7]. Control of these immunosuppressive cells is expected to improve the antitumor effects of nivolumab. It has long been reported that some chemotherapeutic agents specifically modulate the activities of immunosuppressive cells [8]. For example, cisplatin increases the expression of tumor antigens and activates cytotoxic T cells, while 5-fluorouracil (5-FU) and docetaxel reportedly inhibit the activity of Tregs and MDSCs [8]. Paclitaxel has been shown to upregulate the expression of class I major histocompatibility complex proteins in cancer cells, thereby increasing their antigenicity. It also increases the antigen-presenting ability of dendritic cells [8]. Cetuximab, an epidermal growth factor receptor inhibitor, activates cytotoxic T cells by increasing tumor antigens through antibody-dependent cytotoxicity [9]. Therefore, based on these mechanisms, chemotherapeutic agents administered after ICIs may cause immunosuppression in the tumor-bearing host, thereby enhancing the ICI-driven antitumor immune responses [9]. Compared to other chemotherapeutic regimens, PC therapy has been reported to have a significantly higher response rate for head and neck cancer [9]. There are multiple first-line treatment options for recurrent and distant metastatic advanced oral cancer. However, studies have noted that a course of checkpoint inhibitors and subsequent chemotherapy activates an antitumor immune response [9].

Studies on combination immunotherapy have shown that the administration of an ICI alters the immune environment of the host, which is maintained even after switching to other therapies. Based on the findings of the KEYNOTE-048 study [9], the combination of pembrolizumab, an ICI, with platinum and 5-FU, a chemotherapeutic, may be an effective treatment strategy for such cancers.

## Conclusions

Our findings from these two cases of tongue cancer demonstrate that some nivolumab-refractory patients with recurrent or distant metastatic oral squamous cell carcinoma may benefit from subsequent salvage chemotherapy. However, further research is warranted on this topic.

## References

[1] Ferris RL, Blumenschein G Jr, Fayette J (2018). Nivolumab vs investigator's choice in recurrent or metastatic squamous cell carcinoma of the head and neck: 2-year long-term survival update of CheckMate 141 with analyses by tumor PD-L1 expression. Oral Oncol.

[2] Schvartsman G, Peng SA, Bis G (2017). Response rates to single-agent chemotherapy after exposure to immune checkpoint inhibitors in advanced non-small cell lung cancer. Lung Cancer.

[3] Park SE, Lee SH, Ahn JS, Ahn MJ, Park K, Sun JM (2018). Increased response rates to salvage chemotherapy administered after PD-1/PD-L1 inhibitors in patients with non-small cell lung cancer. J Thorac Oncol.

[4] Daste A, De-Mones E, Cochin V, Dupin C, Digue L, Ravaud A, Domblides C (2018). Progression beyond nivolumab: stop or repeat? Dramatic responses with salvage chemotherapy. Oral Oncol.

[5] Ogawara D, Soda H, Iwasaki K, Suyama T, Taniguchi H, Fukuda Y, Mukae H (2018). Remarkable response of nivolumab-refractory lung cancer to salvage chemotherapy. Thorac Cancer.

[6] Chen DS, Mellman I (2013). Oncology meets immunology: the cancer-immunity cycle. Immunity.

[7] Tomihara K, Fuse H, Heshiki W (2014). Gemcitabine chemotherapy induces phenotypic alterations of tumor cells that facilitate antitumor T cell responses in a mouse model of oral cancer. Oral Oncol.

[8] Saleh K, Daste A, Martin N (2018). Response to salvage chemotherapy after progression on immune checkpoint inhibitors in patients with squamous cell carcinoma of the head and neck. J Clin Oncol.

[9] Burtness B, Harrington KJ, Greil R (2019394). Pembrolizumab alone or with chemotherapy versus cetuximab with chemotherapy for recurrent or metastatic squamous cell carcinoma of the head and neck (KEYNOTE- 048): a randomised, open-label, phase 3 study. Lancet.

